# Interrater agreement of two adverse drug reaction causality assessment methods: A randomised comparison of the Liverpool Adverse Drug Reaction Causality Assessment Tool and the World Health Organization-Uppsala Monitoring Centre system

**DOI:** 10.1371/journal.pone.0172830

**Published:** 2017-02-24

**Authors:** Johannes P. Mouton, Ushma Mehta, Dawn P. Rossiter, Gary Maartens, Karen Cohen

**Affiliations:** Division of Clinical Pharmacology, Department of Medicine, University of Cape Town, Cape Town, South Africa; Universita degli Studi del Piemonte Orientale Amedeo Avogadro, ITALY

## Abstract

**Introduction:**

A new method to assess causality of suspected adverse drug reactions, the Liverpool Adverse Drug Reaction Causality Assessment Tool (LCAT), showed high interrater agreement when used by its developers. Our aim was to compare the interrater agreement achieved by LCAT to that achieved by another causality assessment method, the World Health Organization-Uppsala Monitoring Centre system for standardised case causality assessment (WHO-UMC system), in our setting.

**Methods:**

Four raters independently assessed adverse drug reaction causality of 48 drug-event pairs, identified during a hospital-based survey. A randomised design ensured that no washout period was required between assessments with the two methods. We compared the methods’ interrater agreement by calculating agreement proportions, kappa statistics, and the intraclass correlation coefficient. We identified potentially problematic questions in the LCAT by comparing raters’ responses to individual questions.

**Results:**

Overall unweighted kappa was 0.61 (95% CI 0.43 to 0.80) on the WHO-UMC system and 0.27 (95% CI 0.074 to 0.46) on the LCAT. Pairwise unweighted Cohen kappa ranged from 0.33 to 1.0 on the WHO-UMC system and from 0.094 to 0.71 on the LCAT. The intraclass correlation coefficient was 0.86 (95% CI 0.74 to 0.92) on the WHO-UMC system and 0.61 (95% CI 0.39 to 0.77) on the LCAT. Two LCAT questions were identified as significant points of disagreement.

**Discussion:**

We were unable to replicate the high interrater agreement achieved by the LCAT developers and instead found its interrater agreement to be lower than that achieved when using the WHO-UMC system. We identified potential reasons for this and recommend priority areas for improving the LCAT.

## Introduction

Causality assessment of suspected adverse drug reactions (ADRs) is routinely performed in regulatory pharmacovigilance and in epidemiological ADR surveys. Nevertheless, there is no agreement on the best method for assessing causality of suspected ADRs. A 2008 systematic review identified 34 methods to assess ADR causality and grouped these as reaching a decision by global introspection, by following an algorithm, or by methods based on Bayesian probabilistics [1]. Two methods in frequent use are the WHO-UMC system [2], which is an example of a global introspection method, and the algorithm published by Naranjo in 1981 [3].

The WHO-UMC system asks the rater to judge the likelihood of ADR causality into one of six outcome categories, being ‘certain’, ‘probable / likely’, ‘possible’, ‘unlikely’, ‘conditional / unclassified’ (a temporary outcome category, while further information is sought), or ‘unassessable / unclassifiable’. Guidelines have been published to assist the rater, but these are intentionally non-explicit [2]. The Naranjo algorithm consists of ten questions to which a rater responds ‘yes’, ‘no’, or ‘unknown’. Each response is allocated a score, and the sum of scores to the ten questions is usually converted into one of four outcome categories, namely ‘definite’, ‘probable’, ‘possible’, or ‘doubtful’.

**Fig 1:**
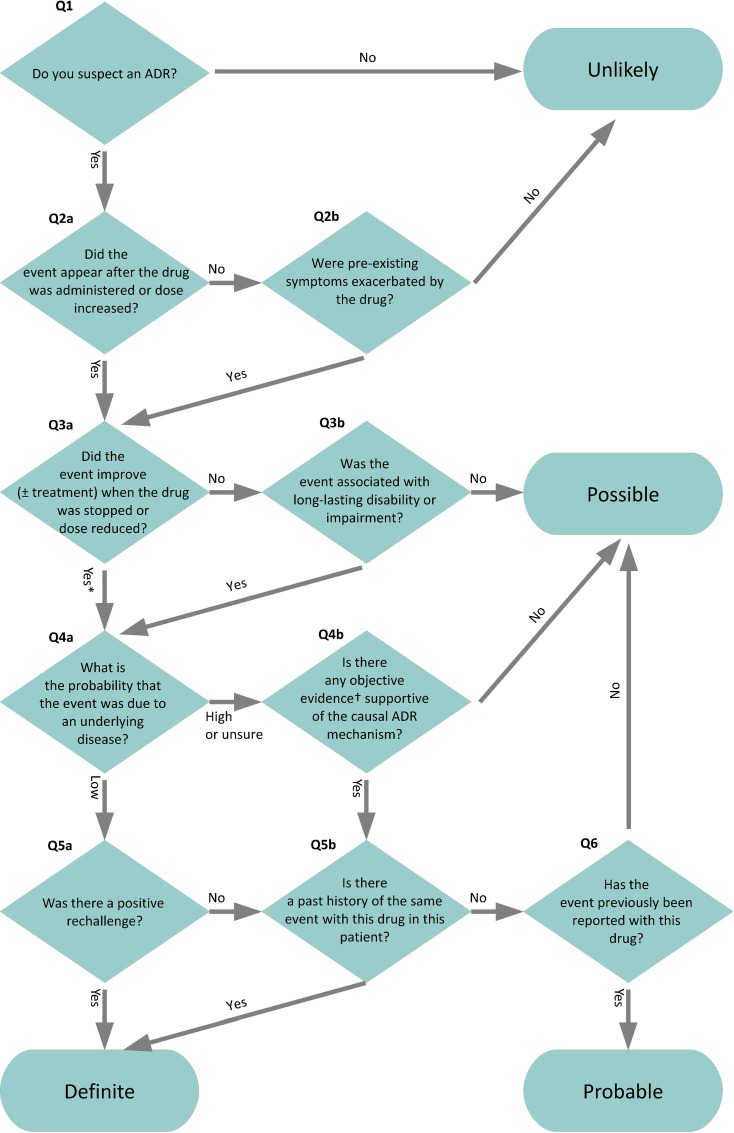
The Liverpool ADR Causality Assessment Tool (LCAT), with numbering of questions as used in this manuscript. * Yes or unassessable. Unassessable refers to situations where the medicine is administered on one occasion (e.g. vaccine), the patient receives intermittent therapy (e.g. chemotherapy), or is on medication which cannot be stopped (e.g. immunosuppressants). †Examples of objective evidence: positive laboratory investigations of the causal ADR mechanism (not those merely confirming the adverse reaction), supra-therapeutic drug levels, good evidence of dose-dependent relationship with toxicity in the patient. LCAT reproduced under a Creative Commons Attribution License. Source: Gallagher [4].

The Naranjo algorithm was adapted into a new assessment algorithm, the Liverpool ADR Causality Assessment Tool (LCAT) [4] by researchers involved in the Adverse Drug Reactions in Children project [5]. The LCAT arranges ten modified questions with their dichotomous responses into a flowchart to arrive at one of four outcome categories: ‘definite’, ‘probable’, ‘possible’, or ‘unlikely’ (Fig 1). The developers of the LCAT found it to result in a more even distribution of outcomes than the Naranjo algorithm, which yielded few ‘definite’ ADRs, and found that the LCAT had higher interrater agreement (IRA) than the Naranjo algorithm, as measured by the kappa statistic [4].

As the LCAT showed early promise of ease of use and high IRA, we considered its use in a prospective survey of ADRs resulting in admission to the medical wards of four hospitals in South Africa, which we have since published [6]. In order to make an informed choice of causality assessment methodology to use in the survey [6], we compared the IRA obtained with the LCAT with that obtained with the WHO-UMC system in a sample of cases from our survey [6]. Secondary aims were to describe the distribution of outcomes achieved with each method, and to investigate whether disagreements on the LCAT outcome could be traced back to specific decision points in the algorithm.

## Methods

### Design

This study was a two-arm IRA study, using a sample of cases identified as suspected ADR-related admissions during our hospital-based survey. In one arm of the study, we measured the IRA achieved when raters used the WHO-UMC, and we measured the IRA achieved when raters used the LCAT on the same cases in the second arm of the study. A novel aspect of our study design was to eliminate the need for a washout period between the two arms by using an incomplete, randomised design. Instead of having each rater assess each case by each method, our design used block randomisation to randomly allocate causality assessment methods to cases and raters, such that each case was assessed by a new random half of raters using one method and by the remaining raters using the second method.

In our survey, we reviewed the folders of all adults admitted to the medical wards of four South African hospitals over a 30-day period [6]. We identified suspected ADR-related admissions with the aid of a screening tool adapted from Rozich [7]. We defined an ADR according to the definition by Aronson and Ferner [8]. We did not regard cases of intentional drug overdose as ADRs. We recorded data from patients’ clinical hospital records; we did not augment missing data from primary care sources. We received ethical approval from the Human Research Ethics Committee of the Faculty of Health Sciences at the University of Cape Town (reference numbers 576/2011 and 828/2015.) The research constituted non‐interventional, anonymised chart review of information documented during routine clinical practice, and did not elicit any information directly from patients. We did not seek individual patient consent. This was approved by the Human Research Ethics Committee.

Four raters were purposefully chosen from our Department to perform the causality assessments. They were a physician/internist (‘Rater 1’), two clinical pharmacists (‘Rater 2’ and ‘Rater 3’), and a clinical pharmacologist (‘Rater 4’), all with extensive experience in various pharmacovigilance roles.

We drew a random sample of ten cases from the 289 admissions flagged as suspected ADR-related admissions in our survey (total 1,951 admissions). We used these ten cases in a self-guided training session on the use of the LCAT and the WHO-UMC system. None of the raters had experience in using the LCAT prior to the training session, but all were familiar with ADR causality assessment by global introspection, although not necessarily with the WHO-UMC system.

We drew a second random sample of 48 cases after excluding the ten cases used in training. We allowed only one randomly selected admission per patient into the sample to avoid clustering of patients re-admitted. Similarly, when more than one drug was suspected to have caused an ADR, one of the suspect drugs was selected at random. Thus, we ended up with 48 unclustered drug-event pairs as samples for this analysis.

Each of the four raters performed causality assessment on the case from a standardised case printout of summary demographic, clinical, laboratory and drug history information in our database. The order of assessment was randomised for each rater to minimise the effect of learning. Communication between raters about the cases was not allowed, but raters were not blinded as to the purpose of their assessments and were allowed unrestricted access to reference sources. All assessments were recorded on a paper case assessment sheet. Raters were requested to trace the path of their assessment process when using the LCAT and to note any problems encountered in order to identify potentially problematic decision points on the LCAT flowchart.

In case no outcome category was achieved on the LCAT (e.g. the rater ‘got stuck’ in the flowchart due to a lack of information to answer a question), or a rater indicated they were unable to choose between two outcome categories, the outcome was coded as ‘unassessable’. We requested raters not to use the ‘conditional’ outcome category on the WHO-UMC system. As a result of these factors, both methods yielded outcomes over five categories: ‘definite’ (the term ‘certain’ was considered equivalent), ‘probable’, ‘possible’, ‘unlikely’, and ‘unassessable’.

### Analysis

We generated a visual representation of each rater’s distribution of outcomes against the mean distribution of outcomes, for each method, as a crude indicator whether our raters exhibited cognitive bias in selecting outcome categories.

To compare the methods’ IRA, we calculated agreement proportions, kappa statistics, and the intraclass correlation coefficient as follows:

1. We defined exact agreement to exist between two raters using the same method if they assessed the case to the same outcome category (including both raters considering the case to be ‘unassessable’), while extreme disagreement was defined to exist if they assessed the case to non-adjacent outcome categories (i.e. one ‘probable’ and the other ‘unlikely’, or one ‘definite’ and the other ‘possible’, or one ‘definite’ and the other ‘unlikely’). Extreme disagreement was also defined to exist where one rater considered the case to be unassessable while the other did not. We calculated the proportion of exact agreement and extreme disagreement between each rater-pair, and we calculated the proportion of specific agreement on each outcome category [9,10].
2. We calculated pairwise Cohen’s kappas among each of the six pairs of raters in an unweighted and a linearly weighted version, for each method. We tested for marginal homogeneity among each rater-pair’s assessments by performing the Stuart-Maxwell chi-square statistic on a cross-tabulation of the two raters’ assessments. We also calculated an overall (‘global’) kappa, for each method, in an unweighted and a linearly weighted version, with 95% confidence intervals constructed by a jackknife technique, using methodology proposed by Abraira [11] suitable for incomplete study designs such as ours. For the weighted kappa calculations, we excluded all cases where one or both raters considered the case to be ‘unassessable’; the weight matrix was thus a 4x4 matrix.
3. We also calculated an intraclass correlation coefficient on each method, using a one-way absolute-agreement single-measures random-effects model (ICC1,1 in the classification of Shrout and Fleiss [12]). We again excluded cases where one or both raters considered the case to be ‘unassessable’ from this calculation.
4. In a sensitivity analysis, we investigated for variability in agreement outcomes when considering ‘unassessable’ ratings as missing data.

To identify potentially problematic decision-making nodes on the LCAT flowchart, we cross-tabulated raters’ total number of positive and negative responses to each question. We also grouped raters into two groups (pharmacists versus clinicians) and cross-tabulated the groups’ total number of positive and negative responses to each question. To interpret these cross-tabulations, we calculated chi-square statistics or performed Fisher’s exact test, as appropriate. Lastly, for each of the LCAT questions we calculated the proportion of specific agreement achieved case-wise between the two raters [9].

Data analysis was performed in Stata version 13.1 (Stata Corporation, College Station, Texas, USA), including the macro ‘kappa2’ [13] to calculate the Abraira kappa and its confidence intervals. We considered p-values <0.05 as significant.

## Results

### Subject characteristics

The 48 sampled patients had a median age of 51.5 years (interquartile range 33.5 to 62.5 years) and 30/48 (63%) were female. 24/48 (50%) were known HIV-infected, and 5/48 (10%) were on treatment for tuberculosis. The sampled drug suspects included antiretrovirals (lamivudine, tenofovir, zidovudine, efavirenz, emtricitabine, and stavudine, totalling 18 cases), cardiac agents (furosemide, hydrochlorothiazide, spironolactone, enalapril, perindopril, and nifedipine, totalling 8 cases), hypoglycaemic agents (5 cases), antituberculosis agents (4 cases), and warfarin (4 cases).

### Distribution of outcomes and investigation for cognitive bias

The 48 cases sampled yielded 96 outcomes on each method (S1 Data). The WHO-UMC system resulted in 8 ‘definite’, 11 ‘probable’, 30 ‘possible’, 34 ‘unlikely’, and 13 ‘unassessable’ outcomes. The LCAT resulted in 3 ‘definite’, 29 ‘probable’, 25 ‘possible’, 32 ‘unlikely’, and 7 ‘unassessable’ outcomes. Individual raters’ distribution of outcomes is given in S1 Table. S1 Fig plots the difference between each rater’s individual distribution of outcomes and the four raters’ mean distribution of outcomes when using the WHO-UMC system; S2 Fig shows identical plots when raters used the LCAT. These plots suggest that, regardless of the causality assessment method used, Rater 2 preferentially assessed cases as ‘probable’, Rater 3 preferentially assessed cases as ‘possible’ and Rater 4 preferentially assessed cases as ‘unlikely’.

### Interrater agreement

Table 1 shows rater-pairs’ cross-tabulated outcomes when using the WHO-UMC system and Table 2 shows their outcomes when using the LCAT.

**Table 1:** Pairwise distribution of outcomes, when using the WHO-UMC system.

	Definite	Probable	Possible	Unlikely	Unassessable
**Unassessable**	0	1	1	1	5
**Unlikely**	0	0	7	13	
**Possible**	0	4	9		
**Probable**	0	3			
**Definite**	4				

**Table 2:** Pairwise distribution of outcomes, when using the LCAT.

	Definite	Probable	Possible	Unlikely	Unassessable	
**Unassessable**	0	1	1	3	1	
**Unlikely**	0	2	9	9		
**Possible**	0	9	3			
**Probable**	1	8				
**Definite**	1				

Using the WHO-UMC system, exact agreement between the two raters occurred in 34 of 48 cases (0.71, range 0.50 to 1.0 among the six rater-pairs). Two rater-pairs (the pair of clinicians and one of the internist-clinical pharmacist pairs) obtained exact agreement on all cases. Using the LCAT, exact agreement occurred in 22 of 48 cases (0.46, range 0.25 to 0.80 among the six rater-pairs, highest for the pair of clinicians). The difference in exact agreement was 0.25 (95% CI 0.025 to 0.47, McNemar’s chi-square p = 0.023). Extreme disagreement occurred with the WHO-UMC system in 3 of 48 cases (0.063) and with the LCAT in 7 of 48 cases (0.15) (Table 3).

**Table 3:** Exact agreement (EA), extreme disagreement (ED), unweighted pairwise Cohen kappa (κ) and linearly-weighted pairwise Cohen kappa (κ_w_) for each of six rater-pairs, using two different causality assessment methods.

Rater-pair	WHO-UMC					LCAT				
	*n*	EA	ED	κ	κ_w_	*n*	EA	ED	κ	κ_w_
**Raters 1 and 2**	7	1.0	0	1.0	1.0	8	0.25	0.13	0.094	0.22
**Raters 1 and 3**	8	0.50	0	0.35	0.53	9	0.33	0	0.13	0.077
**Raters 1 and 4**	6	1.0	0	1.0	1.0	10	0.80	0.10	0.71	0.88
**Raters 2 and 3**	10	0.60	0.10	0.49	0.67	6	0.33	0.17	0.14	0.35
**Raters 2 and 4**	9	0.56	0.11	0.33	0.52	8	0.50	0.13	0.27	0.55
**Raters 3 and 4**	8	0.75	0.13	0.60	0.77	7	0.43	0.43	0.32	0.55

All cross-tabulations were marginally homogeneous, measured by Stuart-Maxwell chi-square statistic. EA: exact agreement. ED: extreme disagreement. κ: unweighted pairwise Cohen kappa. κ_w_: linearly-weighted pairwise Cohen kappa.

The proportion of specific agreement for each outcome category when using the WHO-UMC system was: for ‘definite’ 1.0, for ‘probable’ 0.55, for ‘possible’ 0.60, for ‘unlikely’ 0.76, and for ‘unassessable’ 0.77. The proportion of specific agreement for each outcome category when using the LCAT was: for ‘definite’ 0.67, for ‘probable’ 0.55, for ‘possible’ 0.24, for ‘unlikely’ 0.56, and for ‘unassessable’ 0.29.

Unweighted pairwise Cohen kappas (Table 3) on the WHO-UMC system ranged from 0.33 to 1.0 (mean 0.63, 95% CI 0.31 to 0.95) and on the LCAT from 0.094 to 0.71 (mean 0.28, 95% CI 0.037 to 0.52). The difference in means was 0.35 (95% CI 0.047 to 0.65) and non-zero at p = 0.031. Linearly weighted pairwise Cohen kappas (Table 3) ranged from 0.52 to 1.0 on the WHO-UMC system (mean 0.74, 95% CI 0.51 to 0.98), and from 0.077 to 0.88 on the LCAT (mean 0.44, 95% CI 0.14 to 0.74). The difference in means was 0.31 (95% CI 0.011 to 0.60) and non-zero at p = 0.045.

The overall unweighted Abraira kappa on the WHO-UMC system was 0.61 (95% CI 0.43 to 0.80) and the linearly weighted Abraira kappa 0.73 (95% CI 0.57 to 0.91). On the LCAT, overall unweighted Abraira kappa was 0.27 (95% CI 0.074 to 0.46) and linearly weighted Abraira kappa 0.45 (95% CI 0.25 to 0.67).

The ICC1,1 was 0.86 (95% CI 0.74 to 0.92) on the WHO-UMC system and 0.61 (95% CI 0.39 to 0.77) on the LCAT.

The results of the sensitivity analysis (S2 Table) were similar to those of the main analysis.

### Investigation for potentially problematic questions on the LCAT

We observed only 13 out of the 36 ‘paths’ theoretically possible on the LCAT flowchart among our results. The top three traces occurred 31 times, 21 times, and 20 times, respectively (S3 Table and S2 Data). On two cases raters disagreed on responses to the LCAT questions yet still arrived at the same outcome. Of 59 traces which proceeded beyond Q1 (see Fig 1 for our numbering of the LCAT questions), 35/59 (59%) did not reach Q5a, 29/59 (49%) did not reach Q6, and 27/59 (46%) did not reach Q5b.

Cross-tabulating raters’ positive and negative responses to each question on the LCAT (Table 4), we found disproportionate responses to two questions, Q4a and Q4b. The odds ratio for a positive response on Q4a from a clinician, compared to a pharmacist, was 5.1 (95% CI 1.6 to 16, p = 0.006). Q4b had 22 negative responses and 9 positive responses. Seven of the positive responses came from one pharmacist rater, and there were no positive responses from either clinician rater.

**Table 4:** Distribution of raters’ responses to LCAT questions.

Question ^a^	Negative response ^b^				Positive response ^c^				p-value ^d^
	Rater 1	Rater 2	Rater 3	Rater 4	Rater 1	Rater 2	Rater 3	Rater 4	
1	9	8	4	10	16	14	14	15	0.66
2a	0	0	0	1	16	14	14	14	0.73
2b	0	0	0	1	0	0	0	0	
3a	2	3	1	2	14	11	13	11	0.79
3b	1	0	0	1	1	3	1	1	0.57
4a	6	8	13	4	9	6	1	8	0.008
4b	6	1	11	4	0	7	2	0	<0.001
5a	9	6	1	7	0	0	0	1	0.63
5b	8	13	2	7	1	0	1	0	0.15
6	0	1	0	0	8	12	2	7	1.00

^a^ See Fig 1 for our numbering of questions on the LCAT flowchart.

^b^ Negative responses: ‘High / Unsure’ on question 4a, ‘No’ on all other questions.

^c^ Positive responses: ‘Yes / Unassessable’ on question 3a, ‘Low’ on question 4a, ‘Yes’ on all other questions.

^d^ Chi-square or Fisher’s exact test, as appropriate.

The proportions of specific agreement for each of the LCAT questions are given in S4 Table. Q4a showed the worst agreement (among questions with more than 10 paired responses), with the proportion of both positive and negative agreement on this question lower than 0.70.

Subjectively, our raters commented that they were unclear how to proceed on the LCAT flowchart in situations where (a) the drug was not withdrawn (as opposed to ‘cannot be withdrawn’), (b) the patient died, (c) the patient’s outcome (improved / deteriorated) was different than that of the event being considered, (d) there was a second potential drug cause (the LCAT gives no instruction on dealing with situations when combinations of drugs are implicated in one ADR, and only refers to drugs in the singular), and (e) laboratory results were not available. All four raters also independently reported being ‘channelled’ by the LCAT towards a ‘probable’ outcome when they intuitively considered a case to be ‘definite’. There were three cases (a case of warfarin-associated haematuria, a case of isoniazid-associated liver injury, and a case of insulin-associated hypoglycaemia) where both raters using the WHO-UMC system assessed the case as ‘definite’, while both raters using the LCAT assessed the case as ‘probable’.

## Discussion

We were unable to replicate the high IRA, or the high proportion of ‘definite’ outcomes, reported by the developers of the LCAT. Instead, we found the LCAT’s IRA, and its proportion of ‘definite’ outcomes, to be lower than those achieved by a method of global introspection. We identified potential explanations for the finding of lower IRA by tracing raters’ decisional process on the LCAT flowchart.

To our knowledge, this is the first study which measured the IRA of the LCAT since its development. Previous studies have reported the LCAT’s sensitivity and specificity (against a ‘gold standard’ of consensual expert judgement) as identical to that of the Naranjo algorithm [14]; its distribution of outcomes (in a paediatric hospital-based survey) as similar to that of the Naranjo algorithm [15]; and the exact agreement obtained on the LCAT (between trainee specialists and a predefined ‘correct’ assessment by multidisciplinary panel) as ranging from 20% to 65% [16].

Expert clinical pharmacologists independently assessing ADR case reports by global introspection have been shown to disagree on ADR causality assessment, as well as the presence of drug-drug interactions, the contribution of the ADR to patients’ admission and duration of hospitalisation, the presence of severe morbidity and ADR-related sequelae, and the contribution of the ADR to death [17]. As a result, global introspection has been criticised as ‘neither reproducible, valid, nor accountable’ [18], and it seems intuitive that an explicit, algorithmic method should result in higher IRA than an implicit, subjective method such as global introspection. This, in fact, had been shown by Naranjo et al. in the original publication on the development of their method, which used published case reports to determine IRA: on only an implicit ADR causality assessment comparable to the WHO-UMC system, pairwise unweighted kappa ranged from 0.21 to 0.37, but by applying the Naranjo algorithm, pairwise unweighted kappa improved to range from 0.69 to 0.86 [3].

Since the development of the Naranjo algorithm, however, two studies have compared its IRA to the IRA achieved on a method of global introspection and failed to replicate the higher IRA the Naranjo developers showed. First, in a routine hospital-based ADR surveillance programme, three paediatric clinical pharmacologists achieved similar IRA whether using implicit causality categories or the Naranjo algorithm [19]. A more recent study from the Liverpool group also found similar IRA whether using the WHO-UMC system or the Naranjo algorithm [20]. These studies raised the question whether the Naranjo algorithm is transferable to settings where users are unfamiliar with it or information is less complete than in published case reports.

Similar to the above two studies, which failed to replicate IRA reported by the Naranjo algorithm’s developers, we were unable to replicate the IRA reported by the LCAT developers [4]. Whereas its developers found the LCAT’s linearly weighted pairwise kappas to range from 0.50 to 0.85 and its ‘global’ unweighted kappa to be 0.60 (95% CI 0.54 to 0.67) [4], we found its linearly weighted pairwise kappas to range from 0.077 to 0.88 and its ‘global’ unweighted kappa to be 0.27 (95% CI 0.074 to 0.46).

We found the LCAT’s IRA to be lower than that of the WHO-UMC system using a range of measures. The LCAT’s calculated kappa statistics could be considered ‘fair’ to ‘moderate’ in terms of the frequently-used Landis and Koch interpretation [21], a category lower than WHO-UMC system’s kappa statistics, which were ‘moderate’ to ‘substantial’. Similarly, the LCAT’s ICC (0.61) was a category lower than that of the WHO-UMC system (0.86) in the interpretation of Cicchetti [22] (‘good’ versus ‘excellent’). Moreover, proportions of exact agreement and extreme disagreement among individual rater-pairs, and proportions of specific agreement on each category were consistently higher for the WHO-UMC system than the LCAT.

A possible explanation for the WHO-UMC system’s higher IRA could be the LCAT’s potential for disregarding information that may have otherwise contributed to the decision-making process. Loss of information can firstly occur in any algorithm designed with the potential to bypass some decision-making nodes. As pointed out previously, the LCAT flowchart is structured in such a way that some questions cannot be reached under certain circumstances [14]. We found that potential information on rechallenge and on previous ADRs to the drug was only taken into account in about half of assessments on the LCAT, as those specific questions were simply not reached by raters. Secondly, loss of information can occur in an algorithm when the rater makes a pragmatic choice when ‘the true answer may be somewhere in-between’ the options offered by the algorithm [23]. Our study was not designed to measure how often this may have happened, although our raters anecdotally reported some such instances.

In our study, both causality assessment methods resulted in assessments across all outcome categories. However, the WHO-UMC system resulted in proportionally more ‘definite’ and ‘unassessable’ outcomes, while the LCAT resulted in relatively more ‘probable’ outcomes. This contrasts with the LCAT developers’ finding that ‘definite’ outcomes were most common [4].

The LCAT’s ‘channelling’ towards ‘probable’ outcomes has previously been discussed [14]. It was pointed out that when a rater is unsure about the likelihood that an underlying disease caused the event, she is forced to disregard information about a positive rechallenge (which is generally considered to be a strong indicator of drug causation), as she is steered away from that question in the LCAT flowchart [14]. It was also pointed out that the design of the LCAT requires either a positive rechallenge or a past history of the same event with the same drug in the same patient in order to arrive at a ‘definite’ outcome, and that those conditions are seldom met in routine clinical practice [14].

It would appear that the LCAT, though designed to yield more ‘definite’ outcomes than the Naranjo algorithm, is unlikely to achieve this when using available routine clinical data as was the case in our study design. Nevertheless, in the absence of a gold standard, it remains unclear whether a method with a predilection for ‘probable’ outcomes or one with a predilection for ‘definite’ outcomes should be regarded as preferable.

We postulate some reasons for the differences between our findings and those of the LCAT developers [4]. First, although both studies’ data came from prospective hospital-based ADR surveys, our study population were adults while theirs were children. A number of their patients presented with repeated episodes of febrile neutropenia attributed to cancer chemotherapy; our survey excluded oncology wards but included a large proportion of patients infected by HIV, and antiretroviral therapy was frequently suspected of causing ADRs. Second, there were differences between the raters in the two studies: we used only four raters versus the LCAT developers’ seven, and our raters had a smaller spectrum of expertise. Our raters were not involved in the development of the LCAT, nor were they, before a training session, familiar with its use, and the LCAT developers had shown that IRA on the LCAT increased as their raters’ familiarity with the method grew. Third, our assessments were based on available clinical case data summarised and captured onto a database, and not on raw case data in patient folders or data specifically collected for study purposes. Together with our use of a sensitive but non-specific trigger tool for case identification, this resulted in a high frequency of suspected ADRs ultimately assessed ‘unlikely’ and ‘unassessable’, unlike the LCAT developers’ distribution of outcomes.

In addition to the methodological differences between the studies, we identified two questions in the LCAT flowchart as points of significant divergence. These were Q4a (“What is the probability that the event was due to an underlying disease?”) and Q4b (“Is there any objective evidence supportive of the causal ADR mechanism?”). Answering these two questions requires some clinical judgement, and our pharmacist-raters answered these questions differently from our clinician-raters. This is not a novel finding: Naranjo et al. reported in their original paper that a question on alternative causes of the purported ADR resulted in most disagreement between their raters, with pharmacists answering the question differently to physicians [3]. Another early study tracing two raters’ decision-making process on the Kramer algorithm [24] also found that decision nodes requiring raters to express some form of subjective judgement resulted in disagreement more frequently than decision nodes with ‘factual’ questions [25].

A modification of the LCAT was recently published, in which the modified tool was used in a pharmacogenetic study to phenotypically link one ADR (pancreatitis) to one class of drugs (thiopurines) [26]. This modification inter alia discarded four questions, including Q4b, rephrased four questions, including Q4a, and swapped the order of Q5a and Q4a. While the authors of the modification provided neither a justification for the modification, nor evidence of the modified tool’s validity, and while it is debatable whether the modified tool would be useful outside such a restricted setting, it is tempting to speculate that they may have been faced with some of the same problems on Q4a and Q4b we have found.

We would suggest that improvements to the LCAT may be warranted. Such improvements should aim to reduce the tool’s potential for loss of information, should focus on maximising agreement on the two ‘subjective’ questions identified as sources of disagreement, and should provide guidance on how to answer questions in ambiguous situations.

Limitations of our study include that case summaries may have lacked detailed information, that raters had prior familiarity with global introspection as a methodology but not with the LCAT, and that raters were aware of the purpose of their assessments. We conducted self-guided training in the use of the LCAT, which was not standardised; a training module [16] is still under development by the authors of the LCAT. We used a small convenience sample of raters, limiting the generalizability of our results. It appears that bias existed in raters’ preference for certain outcome categories, which may have influenced our overall kappa estimates and ICC. However, bias was not present when calculating pairwise Cohen kappas (cross-tabulations were marginally homogeneous in all instances). For these reasons further interrater (and intra-rater) agreement studies would be essential to investigate the LCAT’s usefulness, as well as that of any modifications to it.

## Conclusion

Causality assessment of ADRs is an expression of the rater’s uncertainty in assessing the strength of evidence in favour of drug causation, rather than an expression of any inherent quality of the ADR itself. Maximising IRA has therefore been criticised for ‘suppressing’ disagreement, when disagreement per se is valuable information [27]. Nevertheless, IRA remains a widely used way to quantify a method’s repeatability. Our study suggests that causality assessment using the LCAT is less repeatable than a method of global introspection, when it is used outside of the setting in which it was developed.

## Supporting information

S1 Data Interrater agreement comma-separated data (.txt file). Key: tool 1 = WHO-UMC system; tool 2 = LCAT; outcome 1 = definite; outcome 2 = probable; outcome 3 = possible; outcome 4 = unlikely; outcome 5 = unassessable.
(TXT)

S2 Data LCAT paths comma-separated data (.txt file). (TXT)

S1 Fig Difference between raters’ individual distribution of outcomes and the four raters’ mean distribution of outcomes, when using WHO-UMC method. (PDF)

S2 Fig Difference between raters’ individual distribution of outcomes and the four raters’ mean distribution of outcomes, when using LCAT. (PDF)

S1 Table Raters’ distribution of outcomes when using two different causality assessment methods. (PDF)

S2 Table Results of sensitivity analysis. (PDF)

S3 Table Frequency of paths (traces) on the LCAT. (PDF)

S4 Table Case-wise response pairs to LCAT questions, and proportions of specific agreement on LCAT questions. (PDF)
