# Supplementary material for: Interrater agreement of two adverse drug reaction causality assessment methods: A randomised comparison of the Liverpool Adverse Drug Reaction Causality Assessment Tool and the World Health Organization-Uppsala Monitoring Centre system
Source: PLoS One. 2017 Feb 24;12(2):e0172830. doi: 10.1371/journal.pone.0172830 (PMC5325562; doi:10.1371/journal.pone.0172830)

Figure S2. Difference between raters' individual distribution of outcomes and the four raters' mean distribution of outcomes, when using LCAT.

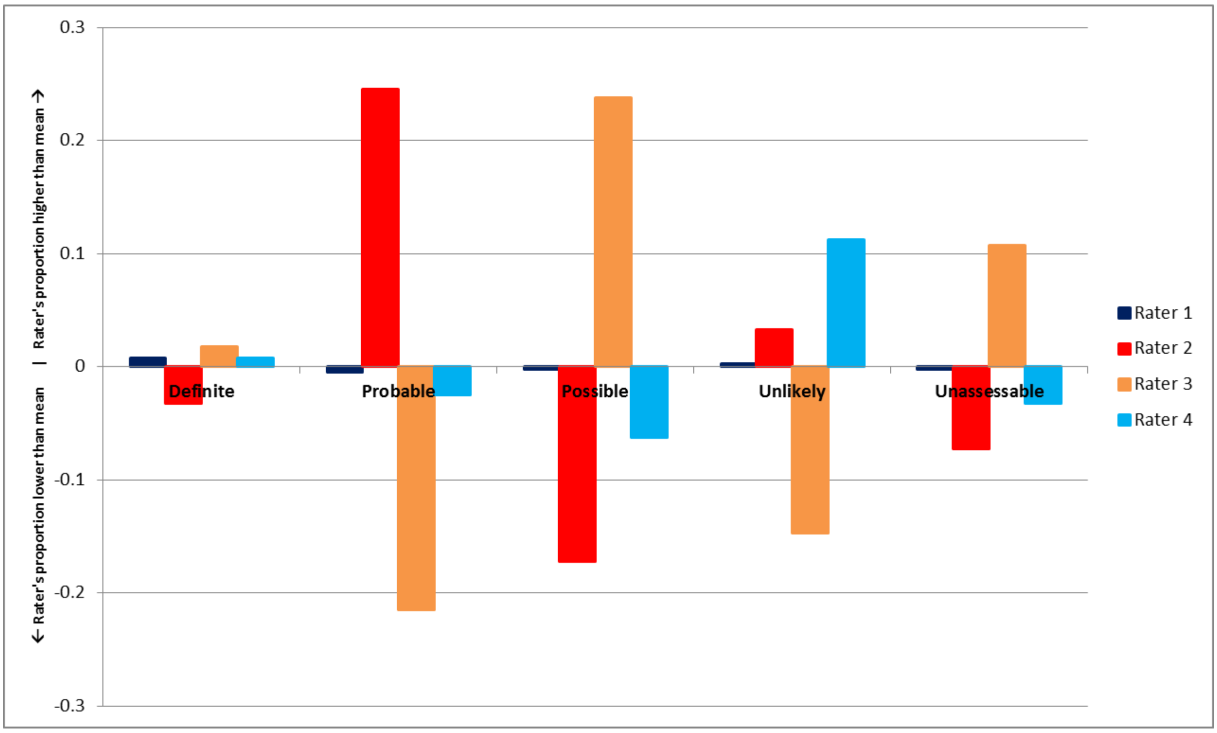

Supplement: S2 Fig — (PDF) [file pone.0172830.s004.pdf]
