# Supplementary material for: Interrater agreement of two adverse drug reaction causality assessment methods: A randomised comparison of the Liverpool Adverse Drug Reaction Causality Assessment Tool and the World Health Organization-Uppsala Monitoring Centre system
Source: PLoS One. 2017 Feb 24;12(2):e0172830. doi: 10.1371/journal.pone.0172830 (PMC5325562; doi:10.1371/journal.pone.0172830)
Supplement: S1 Table — (PDF) [file pone.0172830.s005.pdf]

Table S1. Raters' distribution of outcomes when using two different causality assessment methods.

	Definite	Probable	Possible	Unlikely	Unassessable	Total
**Rater 1 – WHO-UMC**	3 (14%)	2 (10%)	4 (19%)	8 (38%)	4 (19%)	21
**Rater 2 – WHO-UMC**	2 (7.7%)	6 (23%)	8 (31%)	7 (27%)	3 (12%)	26
**Rater 3 – WHO-UMC**	3 (12%)	1 (3.9%)	11 (42%)	8 (31%)	3 (12%)	26
**Rater 4 – WHO-UMC**	0	2 (8.7%)	7 (30%)	11 (48%)	3 (13%)	23
Subtotal: WHO-UMC	***8 (8.3%)***	***11 (11%)***	***30 (31%)***	***34 (35%)***	***13 (14%)***	***96***
**Rater 1 – LCAT**	1 (3.7%)	8 (30%)	7 (26%)	9 (33%)	2 (7.4%)	27
**Rater 2 – LCAT**	0	12 (55%)	2 (9.1%)	8 (36%)	0	22
**Rater 3 – LCAT**	1 (4.6%)	2 (9.1%)	11 (50%)	4 (18%)	4 (18%)	22
**Rater 4 – LCAT**	1 (4.0%)	7 (28%)	5 (20%)	11 (44%)	1 (4.0%)	25
Subtotal: LCAT	***3 (3.1%)***	***29 (30%)***	***25 (26%)***	***32 (33%)***	***7 (7.3%)***	***96***
**Combined total: WHO-UMC and LCAT methods**	**11 (5.7%)**	**40 (21%)**	**55 (29%)**	**66 (34%)**	**20 (10%)**	**192**
