# Supplementary material for: Interrater agreement of two adverse drug reaction causality assessment methods: A randomised comparison of the Liverpool Adverse Drug Reaction Causality Assessment Tool and the World Health Organization-Uppsala Monitoring Centre system
Source: PLoS One. 2017 Feb 24;12(2):e0172830. doi: 10.1371/journal.pone.0172830 (PMC5325562; doi:10.1371/journal.pone.0172830)
Supplement: S2 Table — (PDF) [file pone.0172830.s006.pdf]

S2 Table. Results of sensitivity analysis.

	WHO-UMC system		LCAT	
**Agreement measure**	**Result: main analysis**	**Result: sensitivity analysis***	**Result: main analysis**	**Result: sensitivity analysis***
Exact agreement	34/48 (0.71)	29/40 (0.73)	22/48 (0.46)	21/42 (0.50)
Pairwise exact agreement (range)	0.50 to 1.0	0.43 to 1.0	0.25 to 0.80	0.25 to 0.89
Extreme disagreement	3/48 (0.063)	0/40	7/48 (0.15)	2/42 (0.048)
Pairwise unweighted kappa (range)	0.33 to 1.0	0.20 to 1.0	0.094 to 0.71	-0.04 to 0.82
Overall unweighted Abraira kappa	0.61 (95% CI 0.43 to 0.80)	0.60 (95% CI 0.38 to 0.82)	0.27 (95% CI 0.074 to 0.46)	0.29 (95% CI 0.066 to 0.52)
Proportions of specific agreement	‘definite’=1.0<br>‘probable’=0.55<br>‘possible’=0.60<br>‘unlikely’=0.76<br>‘unassessable’=0.77	‘definite’=1.0<br>‘probable’=0.60<br>‘possible’=0.62<br>‘unlikely’=0.79	‘definite’=0.67<br>‘probable’=0.55<br>‘possible’=0.24<br>‘unlikely’=0.56<br>‘unassessable’=0.29	‘definite’=0.67<br>‘probable’=0.57<br>‘possible’=0.25<br>‘unlikely’=0.62

* In the sensitivity analysis, all ‘unassessable’ ratings were considered missing data.
