# Supplementary material for: Interrater agreement of two adverse drug reaction causality assessment methods: A randomised comparison of the Liverpool Adverse Drug Reaction Causality Assessment Tool and the World Health Organization-Uppsala Monitoring Centre system
Source: PLoS One. 2017 Feb 24;12(2):e0172830. doi: 10.1371/journal.pone.0172830 (PMC5325562; doi:10.1371/journal.pone.0172830)
Supplement: S3 Table — (PDF) [file pone.0172830.s007.pdf]

Table S3. Frequency of paths (traces) on the LCAT.

Path*	Outcome	Frequency (%)
11.1.01.1.	Definite	1 (1.0%)
11.1.1.01.	Definite	1 (1.0%)
11.1.1.1..	Definite	1 (1.0%)
11.1.1.001	Probable	20 (21%)
11.1.01.01	Probable	4 (4.2%)
11.0101.01	Probable	3 (3.1%)
11.011.001	Probable	2 (2.1%)
11.1.00...	Possible	21 (22%)
11.00.....	Possible	2 (2.1%)
11.0100...	Possible	1 (1.0%)
11.1.01.00	Possible	1 (1.0%)
0.........	Unlikely	31 (32%)
100.......	Unlikely	1 (1.0%)
?.........	Unassessable	6 (6.3%)
11.?......	Unassessable	1 (1.0%)

* Paths are represented as the sequence of responses to 10 questions, in the order Q1, Q2a, Q2b, Q3a, Q3b, Q4a, Q4b, Q5a, Q5b and Q6, labelled as per Figure 1 (main text). '1' indicates a positive response ('yes or unassessable' on Q3a, 'low' on Q4a, 'yes' on all other questions). '0' indicates a negative response ('high/unsure' on Q4a, 'no' on all other questions). '.' indicates that the decision point was not reached. '?' indicates that the rater was unsure how to proceed and terminated assessment at that point.
