# Supplementary material for: Interrater agreement of two adverse drug reaction causality assessment methods: A randomised comparison of the Liverpool Adverse Drug Reaction Causality Assessment Tool and the World Health Organization-Uppsala Monitoring Centre system
Source: PLoS One. 2017 Feb 24;12(2):e0172830. doi: 10.1371/journal.pone.0172830 (PMC5325562; doi:10.1371/journal.pone.0172830)
Supplement: S4 Table — (PDF) [file pone.0172830.s008.pdf]

Table S4. Case-wise response pairs to LCAT questions, and proportions of specific agreement on LCAT questions.

**Question†**	**n (number of paired responses to the question)**	**Response pairs***			**Specific agreement on positive response (proportion)**	**Specific agreement on negative response (proportion)**
		**pos/pos**	**pos/neg**	**neg/neg**		
**1**	42	24	10	8	48/58 (0.83)	16/26 (0.62)
**2a**	24	23	1	0	46/47 (0.98)	0/1 (0.0)
**3a**	22	17	4	1	34/38 (0.89)	2/6 (0.33)
**4a**	20	7	9	4	14/23 (0.61)	8/17 (0.47)
**5b**	9	0	1	8	0/1 (0.0)	16/17 (0.94)
**6**	8	8	0	0	16/16 (1.0)	n/a
**5a**	7	0	0	7	n/a	14/14 (1.0)
**4b**	4	0	2	2	0/2 (0.0)	4/6 (0.67)
**3b**	1	1	0	0	2/2 (1.0)	n/a
**2b**	0	n/a	n/a	n/a	n/a	n/a

* ‘pos’ = ‘yes/unassessable’ on Q3a, ‘low probability’ on Q4a, ‘yes’ on all other questions. ‘neg’ = ‘high probability / unsure’ on Q4a, ‘no’ on all other questions. ‘pos/neg’ includes ‘pos/neg’ and ‘neg/pos’ responses. ‘n/a’ = not applicable.

†Numbering of questions on the LCAT as per Figure 1 (manuscript).
